# Morphological and Ontogenetic Skin Color Changes in the American Alligator (*Alligator mississippiensis*)

**DOI:** 10.3390/ani13223440

**Published:** 2023-11-07

**Authors:** Cadre Francis, Amber Hale, Jennifer Berken, Ted Joanen, Mark Merchant

**Affiliations:** 1 Department of Chemistry, McNeese State University, Lake Charles, LA 70605, USA; 2 Department of Biology, McNeese State University, Lake Charles, LA 70605, USA; ahale@mcneese.edu; 3 Department of Mathematical Sciences, McNeese State University, Lake Charles, LA 70605, USA; jberken@mcneese.edu; 4 Louisiana Department of Wildlife and Fisheries, Grand Chenier, LA 70808, USA; memagain2000@yahoo.com

**Keywords:** Alligatoridae, color change, crocodilian, crypsis

## Abstract

**Simple Summary:**

The skin of American alligators became lighter when animals were exposed to light colored environments. This change occurred over 2–3 months, and was caused by increased pigmented cells in the dermal layers of the skin. Alligators hatch with bright vertical yellow and black stipes that presumably function as camouflage in emergent vegetation. However, the yellow stripes gradually fade/darken until alligators become a homogenous dark gray/black color as adults. Because alligators live in more temperate climates than other crocodilians, we propose that the loss of yellow stripes might help alligators thermoregulate more efficiently during colder months. We measured the brightness of stripes on wild alligators of a broad spectrum of sizes from three different latitudinal locations within their natural range: northern (South Carolina), central (south Louisiana/southeast Texas), and southern (South Florida). The alligators in South Carolina lost stripes faster as they grew larger, while the alligators in the middle portion of the range became darker at a slower rate, and animals in south Florida lost their stripes at an even slower rate. The loss of stripes at different rates might allow alligators to be more efficient at thermal regulation in the different areas that they inhabit.

**Abstract:**

To assess skin color change in alligators, we maintained animals in differently lighted environments and also measured skin colors in an ontogenetic series of wild animals. Juvenile alligators maintained in black enclosures exhibited a gradual lightening of skin color when shifted to white enclosures, and these observed changes were reversible. A histological examination of the skins of alligators maintained in dark tanks showed that the dermis exhibited a dense layer of pigmented cells, while samples from the same animals in light environments exhibited a more diffuse pigmented layer. As alligators grow, they exhibit an ontogenetic loss of stripes that may aid in crypsis and predation. Hatchlings have intense black and yellow vertical stripes that darken with age; adults are a more homogenous black/gray color. Since alligators live in temperate climates and adults have lower surface area/volume ratios, which can be detrimental for the absorption of radiant energy, the darker color of larger animals may also aid in thermoregulation. Alligators at the northern end of their range, with colder climates, exhibited darker skin tones, and the ontogenetic extinction of stripes occurred at a more accelerated rate compared to animals in southern, warmer regions, supporting the idea that latitude-dependent ontogenetic color shift has a role in thermoregulation.

## 1. Introduction

Color changes of skin are known to occur in a wide variety of ectothermic animals. There is a broad spectrum of reasons why animals alter skin color, including crypsis, communication (territorial displays, breeding, etc.), and thermal regulation. There are also a variety of mechanisms by which alterations in skin color can occur. Physiologically based mechanisms of skin color modifications are typically mediated by hormones which cause a redistribution of pigments within the melanocytes or melanophores in the epidermal layers of the skin [1]. The production of color in the skins of reptiles, amphibians, and fish varies, but typically includes the involvement of cell layers in the epidermis and dermis. Close interaction between and localization of pigmented cells (xanthophores, erythrophores, and melanophores) as well as structural cells (iridophores) generate the variations in color [2]. Mechanisms of color change can be classified as physiological, which is a short-term, fast response, or morphological, which is a slow, long-term color change. Physiological color change occurs due to differences in hormone levels, while morphological color change can involve changes in both the density and morphology of pigmented cells [1]. Recent studies have shown that members of the families Crocodylidae and Gavialidae can alter skin color rapidly in response to changes in environmental light conditions; however, members of the family Alligatoridae were unable to respond [1]. In the present study, we investigated the possibility that alligators can alter skin color in response to long-term changes in their environment.

Cryptic color patterns on the surface of animals serve a variety of purposes, including the evasion of predators. Hatchling alligators are only approximately 25 cm at the time of hatching, and thus are subject to high predation pressure from wading birds, raptors, fish, mammals such as racoons, and larger alligators [3,4,5]. Alligators are hatched with vivid yellow and black vertical stripes along the entire length of the lateral surface that help these animals blend in with emergent vegetation habitats on which they rely for cover during the first couple of years [6,7]. The skin color of larger alligators is typically much darker, with a substantial darkening of the yellow stripes to create less contrast and sometimes a homogenous gray or black coloration. In addition, camouflage can provide aid to larger alligators as it conceals them from prey, thus potentially making them more effective predators. Larger alligators, particularly males, tend to inhabit more open water environments devoid of emergent vegetation [8], and thus the loss of stripes may make adult alligators less conspicuous in open water habitats and thereby play an important role in predation.

Skin color can also play an important role in the thermal regulation of ectotherms. Bearded dragons (*Pogona vitticeps*) are known to alter skin coloration for the purposes of thermoregulation [9]. In addition, Walton and Bennett [10] demonstrated that two species of Kenyan chameleons (*Chamaeleo dilepis* and *Chamaeleo jacksonii*) became darker during morning basking. Green treefrogs (*Hyla cinerea*) have been shown to alter skin reflectance for the purpose of thermoregulation [11]. However, these changes were all rapid, typically occurring within a few hours, and were mediated by relatively rapid physiological processes dependent on the action of hormones rather than morphological changes in the skin. We analyzed the ontogenetic loss of stripes in different populations of American alligators located at different latitudes to determine if the transition to darker colors may play a role in the thermal regulation of adults.

## 2. Materials and Methods

**Measurement of skin color**. To determine if alligators were capable of skin color change in response to changes in light conditions, we maintained 20 juvenile alligators (70–90 cm) in 1.0 m^2^ covered enclosures at 28.8 °C. They were initially kept in black tanks and then shifted to white enclosures. The alligators were fed a diet of dried pelletized food (Burris Mill and Feed, Franklinton, LA, USA) ad libitum. Their tail scutes were clipped to enable identification for long-term studies. To determine the skin color of alligators, each animal was measured at the same anatomical locations weekly: center of the head, right side of the neck, right flank, and the second yellow tail stripe on the right side. Reflectance of the skin was measured using a calibrated Konica-Minolta CR-410 chromameter. The instrument was held perpendicular to the skin surface and in contact with the skin, with the reflective color measured in the reflected flash from a xenon bulb [1]. The L* term [12] was used to measure the lightness of the skin on a gray scale ranging from 0 (black) to 100 (white). Animals were initially maintained in a dark environment for three weeks, then shifted outdoors to a light (white) 7 m^2^ tank of approximately 50% water and 50% dry surface at environmental temperatures, which was left uncovered. The outdoor enclosure was placed in an area with exposure to full sun and the animals were provided with a 1.0 m^2^ elevated basking platform that was also suitable for use as a shade area underneath. The skin color of each animal was measured weekly until the color stabilized, and then the animals were moved back into the indoor dark environments. The skin color was monitored weekly until the color again stabilized.

To measure ontogenetic changes in color and pattern, wild alligators of a wide variety of sizes at McFaddin National Wildlife Refuge (Texas, *n* = 97, 44.7–297.2 cm), Miami Corporation Grand Chenier, Louisiana (*n* = 114, 40.7–332.7 cm), Everglades National Park (Florida, *n* = 37, 41.7–294.0 cm), and Yawkey Wildlife Center (South Carolina, *n* = 35, 26.0–274.5 cm) were captured at night from a boat using a spotlight. Animals of approximately 1.3 m or less were captured by hand, and animals larger than 1.3 m were captured using a locking cable snare. Two types of snares were used to capture larger animals. In McFaddin NWR and Everglades National Park, cable snares were used. In Yawkey Wildlife Center, larger alligators were captured using baited trip snares. The animals were measured, their sex determined, and the skin color of black and yellow stripes on the tail and flank measured using a Konica-Minolta CR-410 chromameter. The animals were released at the site of capture within 5 min. To measure the skin color of alligators to examine ontogenetic color changes, the first two yellow stripes and the first two black stripes of the anterior side of the flank on the right side of each animal were measured using a calibrated Konica-Minolta CR-410 chromameter. The values of two black stripes were averaged, and that value was subtracted from the average of the two yellow stripes to obtain a ΔL* value. In addition, the first two yellow stripes and the first two black stripes of the anterior, right side of the tail were measured and the ΔL* calculated. To obtain data from a large variety of sizes of alligators at each field location, we collected skin color data from 35, 97, 114, and 36 alligators in Yawkey Wildlife Refuge in South Carolina, McFaddin National Wildlife Refuge in Texas, Miami Alternative site in Louisiana, and the Florida Everglades, respectively. The disparity in the number of animals captured from each site was due to the proximity to the home of the authors, and also the time and effort spent catching a relatively complete ontogenetic series at each site.

**Histology**. Skin samples were collected from alligators maintained in dark or light tanks using sterile, surgical steel four-millimeter punch biopsy tools (PSS Select, New York, NY, USA). The samples were transferred to 10% neutral buffered formalin for 24 h at room temperature, and then transferred to 70% ethanol for storage until processing. The tissues were dehydrated, paraffin-embedded, sectioned to 7 μm thickness, and mounted on charged slides (Fisherbrand© SuperFrost™ Plus, FisherScientific, Hampton, NH, USA). The tissue sections were deparaffinized, coverslipped, and viewed using a Nikon 50i compound microscope under 40× magnification. The images were analyzed using NIS-Elements Nikon image analysis software. The images were converted to gray scale and 500 µm transects, parallel to the orientation of the dermis/epidermis interface and 50 µm deep to the apical surface of the dermis, and were analyzed for optical density. Six hundred densitometric points along the 500 µm transect were collected and the sum of these points was compared to analyses of tissue transects of samples from the same animal maintained for 7 weeks in white tanks in direct sunlight. The results were expressed as the fold increase in gray scale density for each animal, and the means and standard deviations were compared between animals maintained under different light conditions.

In addition to the gray scale densitometric analyses, the average thickness of the pigmented dermal layers was analyzed. The thicknesses were measured on 50 µm transects perpendicular to the dermis/epidermis interface using five transects spaced at 100 µm. The five samples were averaged, and the means ± standard deviations were compared for each animal maintained under different light conditions. The results were expressed as the percentage increase in dermal thickness for each animal under light conditions (relative to dark), and the means ± standard deviations were compared for animals maintained under different light conditions.

**Statistics and controls**. Statistical analyses were performed using Jamovi software (2021) [13] and the EMMEANS [14] and CAR [15] packages in R version 4.1.1 (2021) [16]. Slopes and intercepts of the regression lines were compared separately for the tail and flank position using Analysis of Covariance (ANCOVA) with separate terms for location and total length and an interaction term for location and total length. Assumptions for the ANCOVA analysis were checked using the Kolmogorov–Smirnov test [17], Levene’s test [18], and the Pearson correlation test [19]. All assumptions for both the tail and flank datasets were satisfied. Tukey multiple comparison procedures as part of the EMMEANS package were used to analyze the pairwise differences while controlling the Type I error rate.

## 3. Results

**Figure 1:**
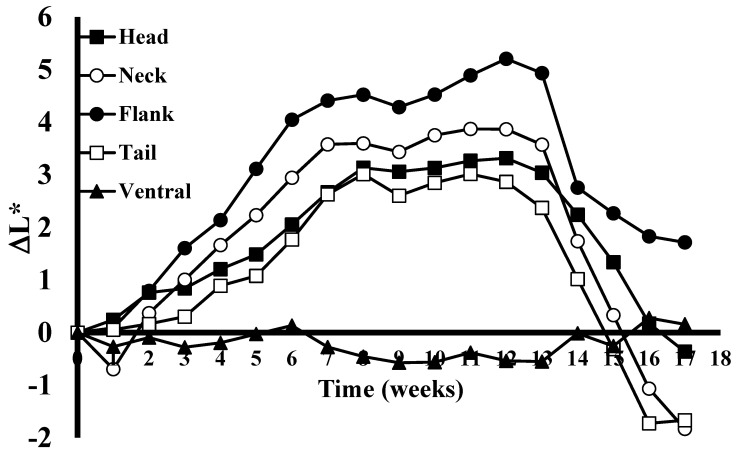
Time-dependent color changes in skin from juvenile alligators maintained in different light conditions. Alligators were placed in an uncovered white tank outdoors for thirteen weeks, then placed in a covered black tank for four weeks. Each data point represents the mean ΔL* value for eight alligators for each anatomical region. The skin colors of the head, neck, flank, and tail areas all changed color to different degrees, but followed the same trend. The ventral surface does not respond to environmental stimuli with color change and thus was used as a negative control measurement.

We found that alligators alter their skin color when shifted from dark to light environments. During the skin color alteration experiment, the maximum skin color change was observed after the juvenile alligators had been maintained in black tanks and then switched to white enclosures for 8 weeks (Figure 1). The yellow stripes on the flank region showed the strongest response (β = 0.692, t(94) = 12.3, *p* < 0.001), followed by the neck (β = 0.589, t(94) = 6.29, *p* < 0.001), head (β = 0.360, t(94) = 15.8, *p* < 0.001), and then tail (β = 0.360, t(94) = 5.99, *p* < 0.001). Rates of mean color change from baseline for the flank and neck position (F(1, 470) = 1.50, *p* = 0.221) and head and tail position (F(1, 470) = 0.00, *p* = 0.997) were the only pairs shown to not be statistically different through week 7. The ventral surface does not change, and was used as a negative control for this study (β = 0.004, t(94) = 0.108, *p* = 0.915). The analysis of the colors of the lateral yellow stripes of the flank showed that this area became progressively lighter each week for the first 8 weeks (Figure 1). The mean color change from baseline increased by 0.692 gray scale units for each additional week. During the initial weeks of the study there was an average difference of approximately 0.67 gray scale ΔL* units/week, which later slowed to approximately 0.24 ΔL* units/week for two weeks (7 and 8) before the lightest observed color was recorded at week 12. During this time frame, the color of the black stripes remained constant. Upon stabilization of the brightness of the yellow stripes, shifting the animals back into a black environment (week 13) resulted in a darkening of the yellow stripes at a rapid rate of 2.15 ΔL* during the first week, and then averaged a reduction of 0.35 ΔL* for the next four weeks (Figure 1).

A Welch’s ANOVA test showed a significant difference in mean color change from baseline between the five locations at week 12, F(4, 26.3) = 52.6, *p* < 0.001. A Games-Howell post hoc test showed that the flank (M = 5.21, SD = 1.62) had a higher mean color change than both the head (M = 3.32, SD = 0.688, *p* = 0.0151) and tail (M = 2.87, SD = 1.97, *p* = 0.0324) at week 12. All anatomical locations differed from the ventral control (all *p* < 0.001).

After alligators were transitioned back to the black enclosure, the neck (β = −1.37, t(94) = −10.1, *p* < 0.001) showed a faster rate of decrease in mean color change than both the head (β = −0.887, t(94) = −9.81, *p* < 0.001), F(1, 506) = 4.95, *p* = 0.027, and flank (β = −0.773, t(94) = −6.55, *p* < 0.001), F(1, 506) = 10.98, *p* = 0.001. There were no statistically significant differences in the rate of change between the remaining non-control location combinations. Head, neck, flank, and tail rates of change differed significantly from the ventral control location (all *p* < 0.001).

**Figure 2:**
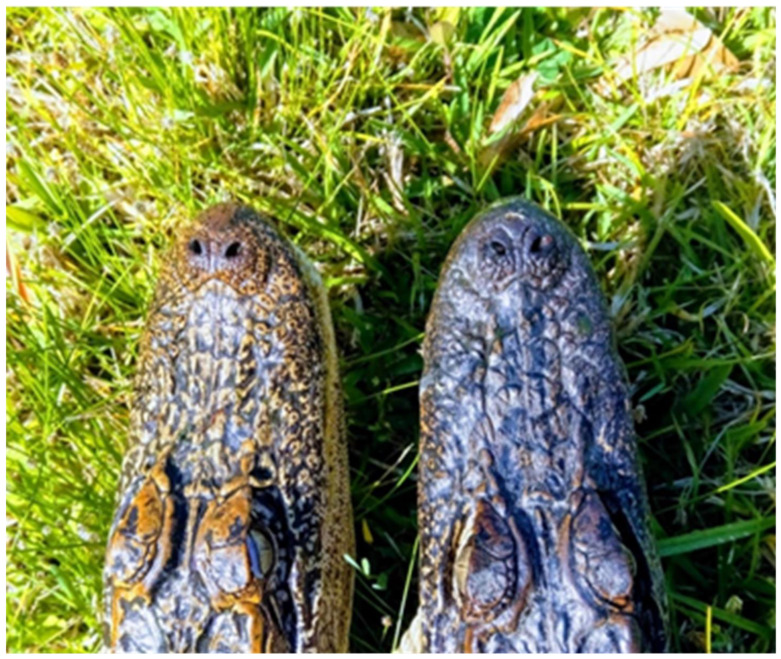
Alligators maintained in light (**left**) or dark (**right**) environments for 6 weeks. When animals maintained in a dark environment are shifted to a light environment, the skin color gradually becomes lighter over the course of several weeks until it stabilizes. A return of the light-skinned alligators back to a dark environment reverses the trend and the skin gradually becomes darker again.

**Figure 3:**
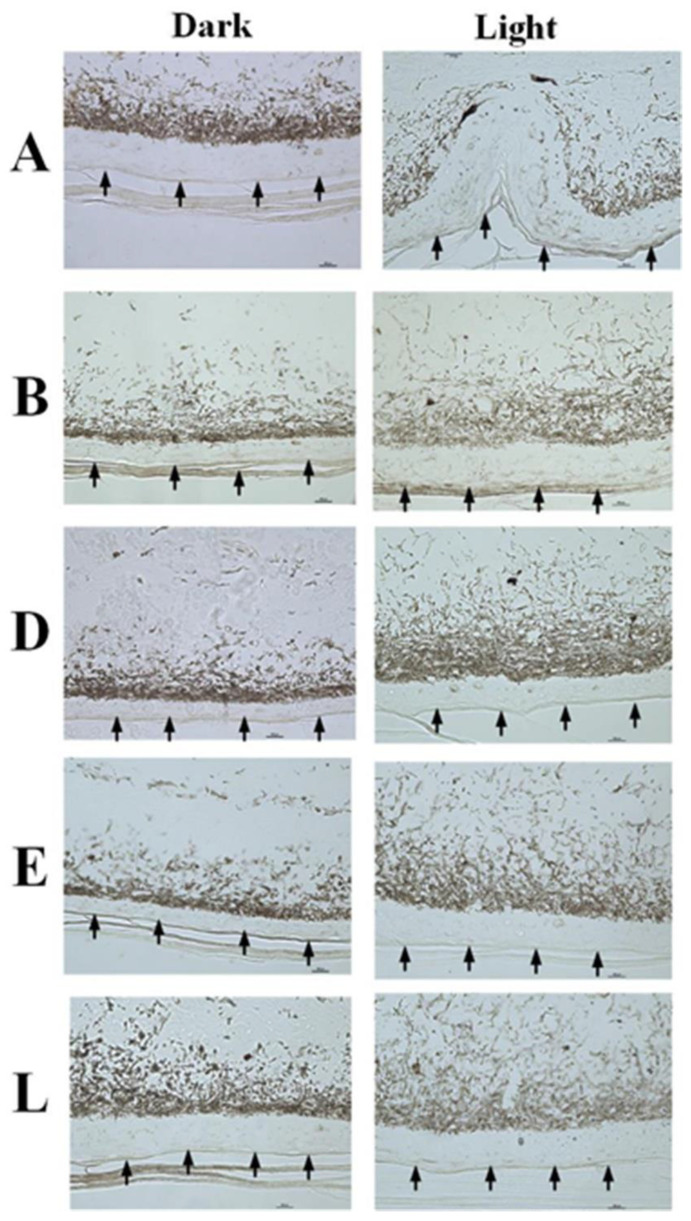
Histological changes in skin color in response to environmental conditions. Skin samples were collected from five alligators that were maintained in dark conditions, and then from the same animals acclimated to white enclosures. Samples from animals kept in the dark revealed that the pigmented cells were clustered toward the apical surface of the dermis, thus causing the skin to appear darker than when the same animals were acclimated to white enclosures. Arrows indicate the apical surface of the epidermis.

**Figure 4:**
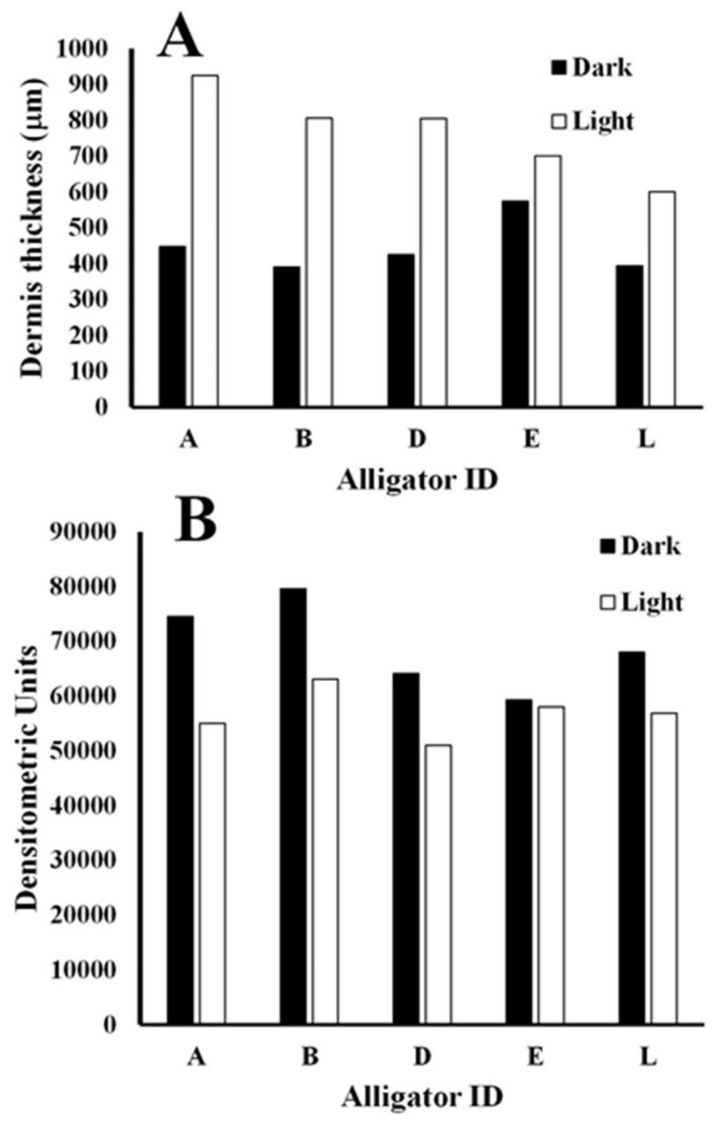
Analysis of histological sections of skin collected from alligators maintained in black or white enclosures. The thickness of the pigmented dermis (**A**) was much greater in alligators maintained in white enclosures, while the density of pigmentation near the apical surface (**B**) was higher in the same alligators maintained in dark enclosures.

In general, the color changes of the neck, flank, head, and tail followed the same pattern for the duration of the 19-week study. The American alligators tested during the study all exhibited a long-term alteration in skin color in response to light and environmental conditions that was abundantly visible to the naked eye (Figure 2). An examination of histological sections showed that the dermis of the skin of animals maintained in light conditions appeared different than that of the same animals in dark conditions (Figure 3). The results of a two-tailed *t*-test of the dermal layer demonstrated that the mean depth of the pigmented layer extended further from the apical surface when animals were maintained in light conditions (t(4) = 4.99, *p* = 0.0001); however, the mean density of the pigmentation (Figure 4B) in the skin of alligators that were maintained in a dark environment (56,789 ± 4426) was higher (t(4) = 3.66, *p* = 0.0044) than that of the same animals when kept in light conditions (69,095 ± 7220).

**Figure 5:**
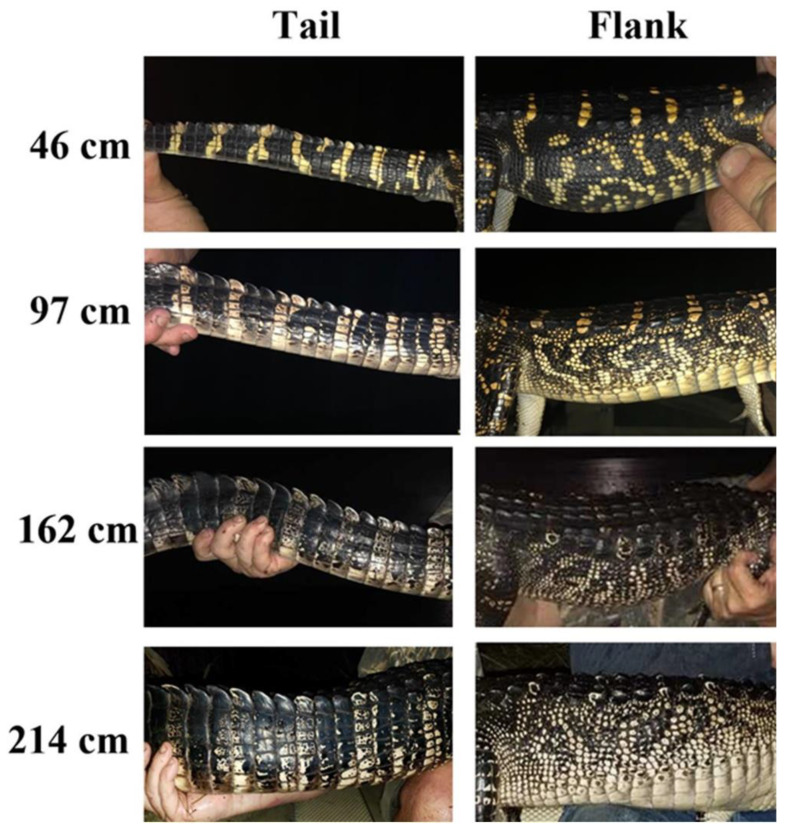
Ontogenetic loss of stripes of alligators from the Gulf Coast of Texas. As alligators grow older and larger, the light-colored stripes become darker. In addition, the lighter stripes become mottled with darker blotches as the animals grow larger.

**Figure 6:**
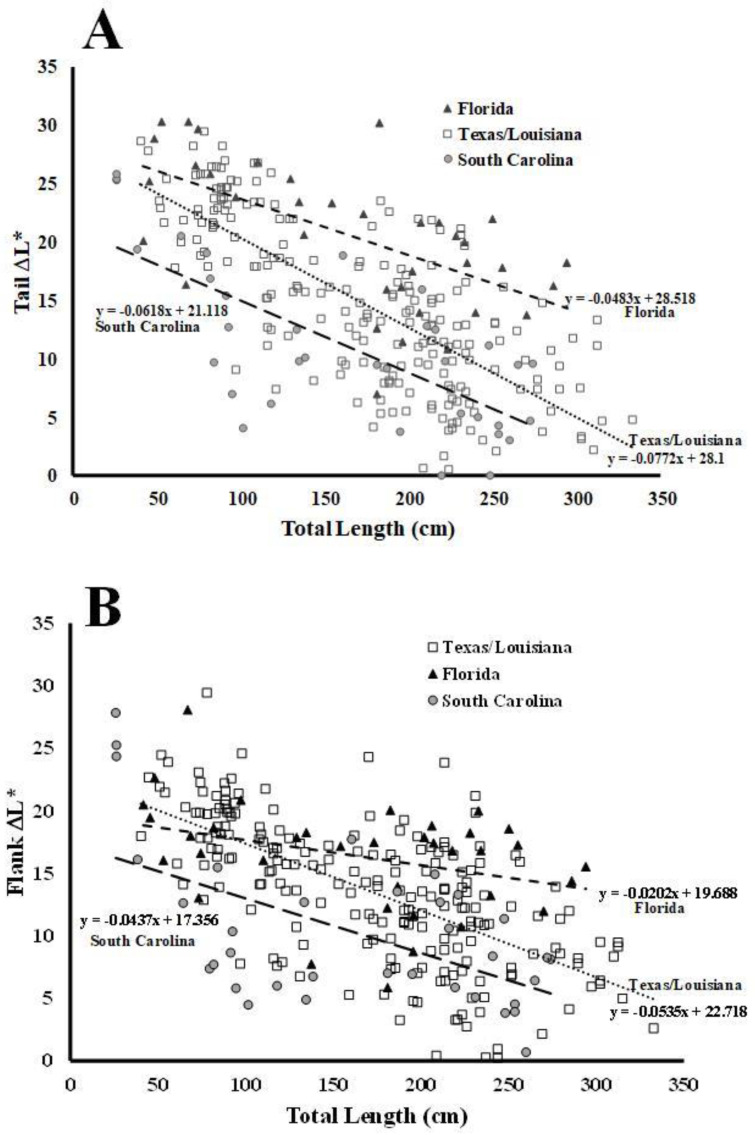
Analysis of the ontogenetic loss of vertical stripes in alligators from Yawkey Wildlife Center on the north coast of South Carolina, the Gulf Coast of southeast Texas and southwest Louisiana, and the Everglades of south Florida. The stripes on the tails (**A**) and flanks (**B**) of alligators become less distinct as the animals grow larger.

The ontogenetic loss of lateral stripes is easy to see with the naked eye (Figure 5). The stripes on hatchlings and alligators up to 100 cm total length are generally very distinct. However, as alligators grow to 150–200 cm the stripes on their tails and flanks become less distinct (Figure 5). As alligators reach lengths of 250–300 cm, the stripes become mottled with black spots and sometimes completely disappear, giving the alligator a homogenous gray color (size class not shown). Results for analyses of the ontogenetic change in color collected for the tail area (Figure 6A) showed that there was a significant difference in the slope of the regression lines by location, F(2, 276) = 3.45, *p* = 0.0332. Tukey post hoc tests [20] showed a statistically significantly smaller negative slope for the ontogenetic color loss of alligators in Florida (β_1_ = −0.0483) compared to those in Texas/Louisiana (β_1_ = −0.0772), t(276) = 2.443, *p* = 0.0401. There was no significant difference in the slopes for animals in South Carolina (β_1_ = −0.0618) compared to animals in either Florida (t(276) = 0.908, *p* = 0.636) or Texas/Louisiana (t(276) = 1.369, *p* = 0.358).

The data showed that the color change of the yellow stripes on the tails of hatchling alligators (25 cm, Figure 6A) in South Carolina is predicted to be darker (ΔL* = 19.6) than that in Texas/Louisiana (ΔL* = 26.2, *p* = 0.0008) or Florida (ΔL* = 27.3, *p* = 0.0033). However, there was no statistical difference in the predicted values of tail ΔL* at 25 cm between Texas/Louisiana and Florida (*p* = 0.8180).

To compare the colors of larger alligators in the different locations, we applied the linear regression analyses to evaluate the predicted values for the tail ΔL* values in alligators 280 cm in length. We chose to analyze the data set at 280 cm because that was the upper end of the sizes of alligators measured at all three locations. The data revealed that the predicted color change for alligators in Florida (ΔL* = 15.0) was higher than that of alligators in southern Texas/Louisiana (ΔL* = 6.5, *p* < 0.0001) or South Carolina (ΔL* = 3.8, *p* < 0.0001). There was no statistical difference in the predicted values of tail ΔL* at 280 cm between South Carolina and Texas/Louisiana, *p* = 0.2085.

Similar results were obtained for the ontogenetic loss of stripes at the flank location (Figure 6B), where a significant difference in the slopes of the regression lines was also observed, F(2, 276) = 4.84, *p* = 0.00862. Again, Tukey’s post hoc tests [20] revealed a difference between the slopes for the alligators in Florida (β_1_ = −0.0202) compared to those in Texas/Louisiana (β_1_ = −0.0535), t(276) = 3.081, *p* = 0.0064. There was no significant difference in the slopes for animals in South Carolina (β_1_ = −0.0437) compared to animals in either Florida (t(276) = 1.732, *p* = 0.195) or Texas/Louisiana (t(276) = 0.953, *p* = 0.607).

Results showed that the predicted flank ΔL* values at 25 cm for alligators in South Carolina (ΔL* = 16.3) differed from the predicted value for those in Texas/Louisiana (ΔL* = 21.4, *p* = 0.0057) but not from the predicted value for Florida (ΔL* = 9.2, *p* = 0.3668). There was no statistical difference in the predicted values of flank ΔL* at 25 cm between Texas/Louisiana and Florida, *p* = 0.4112.

At 280 cm, the predicted value for Florida flank ΔL*, of 14.0, differed from the predicted values for Texas/Louisiana (ΔL* = 7.7, *p* = 0.0001) and South Carolina (ΔL* = 5.1, *p* < 0.0001). There was no statistical difference in the predicted values of flank ΔL* values at 280 cm between South Carolina and Texas/Louisiana, *p* = 0.1659.

## 4. Discussion

Physiological color change is typically rapid and mediated by changes in hormone levels in response to environmental factors [21]. The dispersion or concentration of melanosomes in these cells (integumentary) alter the color display of an organism [22]. However, morphological color change requires more time to establish and is often mediated by a migration of pigmented cells within or between the different layers of the skin [23]. This change involves a change in the structure of the skin, the amount of melanin present in melanophores, and/or the exchange of melanin between cells of the different layers of the skin [24]. Members of the family Crocodylidae rapidly change color with changing light and color environments [1,25]. However, members of the family Alligatoridae do not exhibit this ability [1]. Despite their inability to rapidly change color in response to hormonal cues caused by exposure to light [1], alligators clearly alter their skin color when exposed to light for an extended period of time (Figure 1 and Figure 2). The change is reversible (Figure 1) and seems to be due to changes in the abundance and distribution of the pigmented cells in the dermis (Figure 3). Under conditions of low light, the layer of pigmented cells was dense and compact. In contrast, the skin sections collected from the same animals acclimated to high light environments exhibited a pigmented layer that was more diffuse (Figure 3). This effect is an example of phenotypic plasticity, that allows animals to adjust their skin color to match surroundings and thus reduce the risk of predation or increase the success of prey acquisition [26]. Densitometric analyses showed that the differences in both mean pigment thickness (Figure 4A) and mean density (Figure 4B) were statistically different in the same animals under dark and light conditions.

When alligators hatch, they are highly vulnerable to predation [27]. Predator avoidance is critical for survival [4]. Hatchling and yearling alligators spend the majority of their time in densely vegetated shallow water areas, presumably to avoid detection by potential predators [28]. Larger alligators (≥1.35 m) exhibit cannibalistic activities, and this is thought to be an important factor in the natural population control of alligators, and may account for more than 50% of mortality in hatchlings and yearlings [5]. Therefore, the avoidance of open water areas inhabited by adult alligators might be an important factor for the survival of small alligators [29].

**Figure 7:**
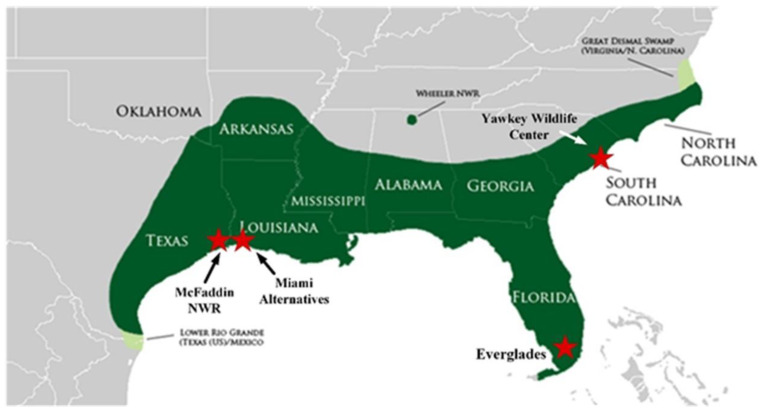
Geographical range of stable populations of alligators. The areas from which skin color data from alligators were collected are indicated.

In addition to light-induced changes in skin coloration, the alligators also exhibited an obvious ontogenetic loss of stripes (Figure 5). The vivid stripes evident in hatchling alligators become less evident in older animals. Alligators are ambush predators, and their foraging behaviors are influenced by environmental conditions [30], and thus a perceived invisibility by potential prey is important for feeding success. Adult alligators tend to spend more time in open water habitats devoid of dense emergent vegetation [29]. Therefore, a shift to a more uniform gray may help larger animals avoid detection in these environments. These changes are due to the yellow stripes becoming darker, as there was no change in the color of the dark stripes. In addition, there was an infiltration of black spots in areas of the yellow stripes, and the yellow stripes became increasingly mottled with darker colors (Figure 5). Large alligators have a much lower surface area/mass ratio than do smaller, younger animals. This relation can be described as S = 13.7 × W^0.64^, where S is surface area and W is equal to the mass of the animal, expressed in grams [31]. The ontogenetic change in surface area/mass is substantial because alligators can increase in mass by several orders of magnitude over their lifetime [32]. As a result, adult alligators heat more slowly than smaller juvenile animals when exposed to solar radiation [33,34]. American alligators live at higher latitudes than any other crocodilian, and wintertime temperatures in this temperate zone often decrease well below freezing for days. Large alligators utilize different mechanisms for heat transfer during different seasons, and it has been proposed that alligators may also have the capacity to acclimatize biochemically to seasonally changing environmental conditions [35,36]. The natural range of alligators spans a large latitudinal area (Figure 7) and extends from the Atlantic coast of northern South Carolina to southern Florida. The wintertime temperatures vary significantly between the northern and southern extremes of this range (Table 1). If part of the function of the ontogenetic shift from bright yellow stripes to a uniform gray/black skin color is to increase the ability to absorb solar radiation for thermoregulation, then we would expect to see differences in the rates of ontogenetic color change in populations at different latitudes. This means that the ontogenetic shift of the lighter yellow stripes to darker gray colors might be important for the increased thermoregulatory capacity of larger alligators. This idea of latitude-dependent coloration in alligators is supported by the data displayed in Figure 6. For alligators in the size range of hatchlings (approximately 25 cm), the yellow stripes on the tails (Figure 6A) from South Carolina were significantly darker than those from the more southern and warmer location of the Florida Everglades. In addition, the coloration of the vertical stripes on the flank of animals from South Carolina was also darker than those of alligators from both the Texas/Louisiana and Florida regions. Furthermore, the ontogenetic loss of stripes on the tail of alligators from South Carolina and Texas/Louisiana was much more rapid than for alligators in Florida. This is presumably due to the warmer winter temperatures in southern Florida (Table 1) providing more opportunities for thermal regulation during the winter, thus making the rapid ontogenetic loss of stripes to darker colors less necessary for animals in this region.

Similarly, the predicted color change of flanks of smaller alligators (25 cm) in South Carolina was darker than those of animals from the lower latitudes of Texas/Louisiana and Florida (Figure 6B). Because the L* values for the black stripes were the same in virtually all alligators at all anatomical locations, this means that the yellow stripes were darker in the northern reaches of the range of the alligator (South Carolina). The predicted changes in flank stripes of the alligators from both South Carolina and the Texas/Louisiana region were both darker than those of alligators from Florida at 280 cm. Furthermore, the ontogenetic loss of stripes occurred at a much lower rate in tail stripes in alligators in Florida compared to those in Texas/Louisiana and South Carolina. This is presumably due to the much warmer temperatures in southern Florida compared to the other regions of this study (Table 1). The average daytime high temperatures during the winter months in the Florida Everglades are 10–12 °C higher than those at Yawkey Wildlife Refuge in South Carolina (Table 1). The rapid loss of yellow stripes likely occurs to allow alligators in this region to become darker more rapidly as they grow, to compensate for a decreasing body surface area/mass. This would allow more efficient thermal regulation in regions where the winter temperatures are lower for a longer period of time. Also, a rapid change of color would not be as important for alligators that inhabit the more southern latitudes (Florida), where temperatures are warmer and there are more opportunities for effective thermal regulation by basking. The differences in the winter high temperatures between the study sites in South Carolina and Texas/Louisiana were much smaller (1–2 °C, Table 1), and thus the differences in the skin colors were less substantial for alligators that live in these regions (Figure 6). Taken together, these data provide strong evidence for differential skin colors and rates of the ontogenetic loss of stripes based on latitudinal temperatures and opportunities for thermal regulation.

## 5. Conclusions

The morphological changes in skin coloration described in this study represent the first description of color change in response to environment in a member of the family Alligatoridae (Figure 1). The alteration in color is due to changes in the densities of pigmented cells in the dermal layer of the skin (Figure 3) and occurs over long periods of time (weeks). The ontogenetic loss of vertical stripes occurs over decades and is easily visible (Figure 5). This ontogenetic shift in color is latitude-dependent (Figure 6), and most likely represents geographically different kinetics of color change due to different climates (Table 1). Alligators in colder climates lose light yellow stripes faster to become more efficient in the absorbance of solar radiation. This is the first description of color change in crocodilians for the purpose of thermal regulation.

## Figures and Tables

**Table 1:** Average winter temperatures in all locations where data were collected during the study. This table shows the high and low temperatures in the locations and can be used to provide context to explain the differences in the ontogenetic color shift of alligator populations in these locations. (National Oceanic and Atmospheric Administration; National Weather Service).

	Average Low/High Winter Temperatures (°C)		
	Everglades National Park, Homestead, Florida 25°45′45 N, 80°43′46 W	McFaddin NWR, Sabine Pass, TX 29°42′10 N, 94°05′23 W	Yawkey Wildlife Center, Georgetown, South Carolina 33°13′34 N, 79°11′59 W
December	14/26 °C	11/17 °C	7/16 °C
January	12/25 °C	8/15 °C	5/13 °C
February	13/27 °C	11/17 °C	6/15 °C

## Data Availability

Data sets are available upon request.
